# Long-term effects of pneumonia in young children

**DOI:** 10.15172/pneu.2015.6/671

**Published:** 2015-12-01

**Authors:** Keith Grimwood, Anne B. Chang

**Affiliations:** 113 Menzies Health Institute Queensland, Griffith University, Gold Coast, Queensland Building G40, Gold Coast campus, 4222 Australia; 213 Department of Infectious Disease and Immunology, and Department of Paediatrics, Gold Coast Health, Gold Coast, Queensland, Australia; 313 Queensland Children’s Medical Research Institute, Queensland University of Technology, Brisbane, Queensland Australia; 413 Menzies School of Health Research, Charles Darwin University, Darwin, Northern Territory Australia; 513 Department of Respiratory and Sleep Medicine, Lady Cilento Hospital, Brisbane, Queensland, Australia

**Keywords:** asthma, bronchiectasis, child, chronic obstructive pulmonary disease, pneumonia

## Abstract

Each year an estimated 120 million episodes of pneumonia occur in children younger than 5 years of age, resulting in one million deaths globally. Within this age group the lungs are still developing by increasing alveoli numbers and airway dimensions. Pneumonia during this critical developmental period may therefore adversely affect the lung’s structure and function, with increased risk of subsequent chronic lung disease. However, there are few longitudinal studies of pneumonia in otherwise healthy children that extend into adulthood to help address this important question. Birth cohort, longitudinal, case-control and retrospective studies have reported restrictive and obstructive lung function deficits, asthma, bronchiectasis, and chronic obstructive pulmonary disease. In particular, severe hospitalised pneumonia had the greatest risk for long-term sequelae. Most studies, however, were limited by incomplete follow-up, some reliance upon parental recall, risk of diagnostic misclassification, and potential confounders such as nutrition, social deprivation, and pre-existing small airways or lungs. More long-term studies measuring lung function shortly after birth are needed to help disentangle the complex relationships between pneumonia and later chronic lung disease, while also addressing host responses, types of infection, and potential confounding variables. Meanwhile, parents of young children with pneumonia need to be advised about the importance of symptom resolution, post-pneumonia. In addition, paying attention to factors associated with optimising lung growth such as good nutrition, minimising exposure to air pollution, avoiding cigarette smoke, and decreasing the risk of preventable infections through good hygiene and having their children fully vaccinated should be emphasised. Finally, in the developing world and for disadvantaged communities in developed countries, public health policies leading to good quality housing and heating, hygiene, education, and improving socio-economic status are also essential.

## 1. Introduction

### 1.1 The burden of pneumonia

Pneumonia is the greatest cause worldwide of childhood morbidity and mortality [1]. The most important respiratory pathogens implicated in severe and fatal cases of childhood pneumonia are *Streptococcus pneumoniae* [2], *Haemophilus influenzae* type b [3], respiratory syncytial virus (RSV) [4], and seasonal influenza virus [5], three of which are already targeted by vaccines that are licensed currently. In 2010, there were an estimated 120 million episodes of clinically diagnosed pneumonia involving children younger than 5 years of age globally, including 14 million with severe disease requiring hospital treatment [6]. Of greater concern, in 2013 as many as one million children died from pneumonia, accounting for 15% of all fatalities in this age group [7]. Most cases (>98%) of pneumonia and pneumonia-related deaths (>99%) are in developing countries, occurring mostly out of hospital and in the first year of life [6]. Nevertheless, pneumonia is also important in developed countries, and prior to the introduction of pneumococcal conjugate vaccines there were an estimated 2.6 million cases of pneumonia annually in children aged younger than 5 years, including 1.5 million requiring hospitalisation, and 3,000 deaths [8]. However, despite high vaccine uptake, disadvantaged indigenous children living in these affluent nations still have amongst the highest rates of pneumonia recorded [9,10]. In addition to the usual recognised risk factors for pneumonia, such as household overcrowding, low birthweight and exposure to inhaled environmental toxicants, indigenous children living in remote communities also experience early and heavy nasopharyngeal colonisation by pneumococcal serotypes not included in conjugate vaccines [11].

### 1.2 Clinical gaps

Despite the high prevalence of pneumonia, including its global clinical and economic importance, many gaps remain in our understanding and management of pneumonia in young children. These gaps, recently summarised in several reviews [12–14], include the long-term effects of pneumonia and its potential role in adult-focused pulmonary disorders [15]. This information is important, for not only managing individual patients, but also for understanding the true burden of disease, including the contribution of early childhood pneumonia to the estimated 200 million people with chronic obstructive pulmonary disease (COPD) and the 235 million people with asthma worldwide [16]. Only then can the full benefit of interventions, such as duration and type of antibiotics or the introduction of new vaccines into national immunisation programmes, be understood. In this review, we focus on the long-term (>6 months) consequences of pneumonia in children and we exclude studies that relate primarily to wheezing illness in children.

## 2. Pneumonia in young children and subsequent adult lung disease

Throughout the last three decades evidence has emerged suggesting that adult-onset chronic pulmonary disorders are likely to have their origins in early life. Here we review the evidence of the published data.

There are two published systematic reviews on the long-term impact of childhood pneumonia [17,18], with one review [17] also including studies relating to bronchiectasis. As the authors used different search terms, there were both similarities and differences between the two reviews. Nevertheless, the Thomson et al [17] review involved two additional prospective studies [19,20] on radiographically confirmed pneumonia.

In the studies included in the review by Edmonds et al [18], a total of 722 children from 13 studies (10 hospital, 3 community-based birth cohorts) were followed for sequelae, 61% of whom were younger than 2 years of age at the time of their pneumonia, and none had any identifiable pre-existing risk factors for chronic pulmonary disorders. Radiographic confirmation of pneumonia was available only for the 379 (52%) children included in the hospital-based studies. The median length of follow-up was 10.8 (interquartile range [IQR] 2.1–17.0) years and overall there was a median 34% (IQR 12%–45%) attrition rate of study subjects. Seventy-seven cases (10.4%; 95% confidence interval [CI] 5.4%–15.4%) were found to have major sequelae involving restrictive lung disease, obstructive lung disease or bronchiectasis. Meta-analysis showed the risk of one of these complications was higher in hospitalised (13.6%; 95% CI 6.2%–21.1%) than non-hospitalised children (5.5%; 95% CI 2.8%–8.3%), whilst among the pathogens, adenovirus pneumonia was associated with the highest risk (54.8%; 95% CI 39.2%–70.5%) of developing one of these major sequelae. Of the sequelae, restrictive lung disease was the most commonly detected (5.5%; 95% CI 2.5%–10.2%). Bronchiectasis, either alone (0.9%; 95% CI 0.07%–8.7%) or in combination with restrictive lung disease (1.2%; 95% CI 0.05%–7.7%), was found only in those who had been hospitalised for pneumonia and obstructive lung disease in those with a history of severe adenovirus pneumonia (2.8%; 95% CI 0.18%–6.4%). Nevertheless, in this review lung function tests suggesting airway obstruction were also reported in up to one-third of 62 South African children 1–7 years after their pneumonia [21] and in 14 of 18 (78%) 7–8-year-old children with a history of chlamydial pneumonia in early infancy [22]. Although children younger than 2 years of age had a greater risk of sequelae than those aged 2–5 years at the time of their pneumonia, this did not reach statistical significance (13.4%; 95% CI 4.5%–22.3% vs 8.7%; 95% CI 3.1%–14.3% respectively, odds ratio [OR] 1.22 [95% CI 0.21–7.1]). Furthermore, in the multivariable model only hospitalisation remained a major risk factor for sequelae (OR3.65 [95%CI 1.96–6.8]).

Here, we summarise studies identified from these reviews and our own search with a focus on prospective and case-control studies. Relevant studies were identified by searching the PubMed database using the terms ‘children’ and ‘pneumonia’ and ‘long term’, respectively in their titles or abstracts without language restrictions. Studies published prior to 13 April 2015 were included.

### 2.1 Prospective longitudinal and case-control studies

These data are summarised in Tables 1 and 2. Three large follow-up, community-based, birth cohort studies from the United Kingdom (UK) assessing sequelae in non-hospitalised children with clinically diagnosed pneumonia were identified. The extracted information for two birth cohort studies was from health visitor records of infants born between 1920 and 1930 in Hertfordshire County, England (males only) [23], and between 1921 and 1935 in Saint Andrews, Scotland, respectively [24]. The surviving participants from both cohorts were evaluated when they were aged in their 50s and 60s. The third community-based birth cohort study was from the 1958 British National Child Development Study where, at the age 7-year follow-up interview, parents were asked if their child ever had pneumonia [25]. Subjects in this study were assessed at 34 to 35 years of age. Each of these three birth cohort studies reported that pneumonia in early childhood was associated with restrictive lung function.

**Table 1:** Prospective birth cohort studies examining the effects of childhood pneumonia upon adult lung function

Study	Country	Year(s) of inception	Year(s) of study	Source of diagnosis	No. with pneumonia	No. with pneumonia studied (%)	No. without pneumonia studied	Ages (years)	Median follow-up (years)	Main findings
Barker et al. [23]^a^	UK	1920–30	1989	Health visitors prospective reports of illness in the first yr, and between 1 and 5 yrs of life	122^b^	122 (100)	703	59–67	60	Bronchitis or pneumonia in male infants was associated with significantly reduced adult values for FEV_1_ (−0.17 L; 95% CI −0.32, −0.02) and FVC (−0.24 L; 95% CI −0.24, −0.07) and increased odds of wheezing (OR 1.83 [95% CI 1.05, 3.20]) independent of smoking, birth weight and social class.
Shaheen et al. [24]^a^	UK	1921–35	1985–86	Doctors’ records for the first 5 yrs of life	18	10 (53)	229	57.6 ± 4.3^d^	50	Pneumonia in the first 2 yrs of life was associated with significantly reduced adult values for FEV_1_ (−0.39 L; 95% CI −0.67, −0.11; *p* = 0.007) and FVC (−0.60 L; 95% CI −0.92, −0.28); *p* < 0.001) independent of age, sex, height, smoking and other illnesses before age 2 yrs.
Johnston et al. [25]^a^	UK	1958	1992–93	Parents report when child aged 7 yrs	350	193 (55)	1,199	34–35	30	History of pneumonia in the first 7 yrs of life was significantly associated with reduced adult FEV_1_ (−0.102 L; 95% CI −0.175, −0.029; *p* = 0.006) and FVC (−0.173 L; 95% CI −0.243, −0.067; *p* = 0.001) without a change in FEV_1_/FVC ratios, which were unaltered by bronchodilators and were independent of a history of wheezing.
Shaheen et al. [26]	UK	1917–22	1990–91	Health visitors’ reports of illness collected 3 monthly in first yr, then once yearly	20	20 (100)	598	67–74	70	In men only, pneumonia before age 2 yrs was significantly associated with lower adult FEV_1_ (−0.65 L; 95% CI −1.02, −0.29, *p* < 0.001), FVC (−0.51 L; 95% CI −0.94, −0.08; *p* = 0.02) and FEV_1_/FVC ratios (−10.9%; 95% CI −16.9, −4.8; *p* < 0.001) independent of smoking and asthma.
Chan et al. [27]	USA	1980–84	1996–2003	Clinical with CXR (infiltrates, bronchopneumonia or pneumonia) in the first 3 yrs of life	66	44 (67)	308	26–29	26	Those with pneumonia before 3 yrs of age had significantly lower FEV_1_/FVC ratios (−3.23%; *p* = 0.034)), FEF25-75 (−0.508 L/s; *p* = 0.02) and FEF25-75/FVC ratios (−11.14%; *p* = 0.024) at 26 yrs of age, independent of wheeze and smoking, and which were only partially reversible with bronchodilators. Pneumonia was also associated with a two-fold increased risk of actively managed asthma at age 29 yrs (OR 1.95 [95%CI 1.11, 3.44]; *p* = 0.02).
Lopez-Bernal et al. [28]	UK	1972–74	1997	Study team or maternal report of respiratory illness in the first 5 yrs of life^c^	NR	NR	NR	25.0 ± 0.7^d^	25	679 (71%) of original cohort underwent lung function testing. After adjusting for potential confounding factors, lower respiratory tract infections in the first yr of life were negatively associated with all spirometry values, except for FVC, and showed a significant dose-response effect where a two-fold increase in lower respiratory tract infections was associated with reduced FEV_1_ (−0.078 L; 95% CI −0.153, −0.03), and FEV_1_/FVC ratios (−1.23%; 95% CI −2.22, −0.25).
Colley et al. [29]	UK	1946	1966	Parent questioned when child aged 2 yrs (pneumonia, bronchopneumonia or bronchitis)	820	820 (100)	2,502	20^e^	20	In ‘never smokers’, prevalence of day/night cough was 9.1% in group with chest infections vs 5.2% without. In smokers, values were 16.5% and 13.5%, respectively. Difference between groups (with and without chest infections) was significant (p < 0.025). Social class and air pollution had no significant effect.

CI, confidence interval; CXR, chest radiograph; FEV_1_, forced expiratory volume in one second; FVC, forced vital capacity; FEF25-75, forced expiratory flow between 25% and 75% of FVC; NR, not reported; OR, odds ratio; UK, United Kingdom; USA, United States of America

^a^Included in review by Edmonds et al. [18]

^b^Males only

^c^Supplemented by a doctor’s diagnosis when available

^d^Mean ± Standard Deviation

^e^Cohort was born in the last week in March 1946. In 1966, they were all sent a questionnaire at the same time

**Table 2:** Case-control and follow-up studies examining the effects of childhood pneumonia upon adult lung function

Study	Type of study	Country	Year(s) of inception	Year(s) of study	Source of diagnosis	No. with pneumonia	No. with pneumonia studied (%)	No. without pneumonia studied	Ages (years)	Median follow-up (years)	Main findings
Tennant et al. [30]^a^	Case-control (done in adolescence and again in adulthood)	UK	1947	1961–62, 1996–98	Doctor or study team diagnosis of severe lower respiratory illness before 5 yrs of age	167	167 (100)	85	14 49–51	14 50	Severe lower respiratory illness before age 5 yrs was independently associated with lower FEV_l_ at 14 yrs of age (*p* = 0.004) and a greater overall decline in FEV_1_ between 14 to 51 yrs of age (*p* = 0.047).
Mok & Simpson [31]^b^	Case-control	UK	1971–74	1978–81	Infants hospitalised with CXR documented	51	51 (100)	51^c^	7.20 ± 0.44^d^	7	Ongoing respiratory symptoms in the previous year were significantly higher in the pneumonia group than controls. This included wheeze (*p* < 0.01), chest infections (*p* < 0.001), using medications (*p* < 0.001) and school missed from respiratory illness (*p* < 0.01). Study also had 102 children hospitalised with bronchiolitis and 44 with bronchitis. Compared with controls, those with bronchiolitis had significantly lower FEV_1_ (*p* < 0.05), but not FVC values. Although findings were similar in pneumonia group, the difference was not statistically significant.
Eastham et al. [19]^b^	Controlled follow-up study	UK	1995–98	NR	Clinical with CXR confirmation aged 5–16 yrs	159	103 (65)	248^e^	NR	5.6 (4.4–7.4)^e^	Pneumonia is independent risk factor for persistent cough (OR 2.9 [95% CI 1.45, 5.71; *p* = 0.02) and asthma (OR 4.8 [95% CI 1.43, 16.34]) in cases with non-atopic parents. Compared to controls, cases with pneumonia and atopic parents had significantly lower FEV_1_% pred (−7.0%; 95% CI −10.5, −3.21; *p* < 0.001) and FVC % pred (−4.4%; 95% CI −8.0, −0.78; *p* = 0.017).
Pulchalski et al. [32]^b,f^	Case-control	Gambia	1992–94	NR	Children aged <5 yrs with severe pneumonia	190^g^	68 (36)	67^h^	12–14	13	No significant difference found between cases and controls. 14 of the 83 traced had died of whom 78% died within days to weeks of discharge.
Piippo-Savolainen et al. [33]^f^	Case-control	Finland	1981–82	2000	CXR-confirmed hospitalised children aged 1–24 mths	44	34 (77)	45	18–21	17	No significant difference between groups for lung function, diagnosis of asthma, or prolonged cough in last 12 mths. Study also enrolled infants with bronchiolitis (*n* = 53) group that significantly differed from controls for asthma symptoms (OR 5.07 [95% CI 1.66, 15.50]) and abnormal lung function (OR 4.13 [95% CI 1.36, 12.51]).
Wesley et al. [21]^b,f^	Follow-up of hospitalised cohort	South Africa (‘Black children’)	NR	NR	CXR-confirmed, aged 6–119 mths, but most were from virus or measles	NR	62	0	NR	7	40% had persistent respiratory symptoms (wheeze or cough), 34% with abnormal lung function (8% with obstruction, 16% undefined).
Kycler et al. [20]^b^	Follow-up of hospitalised cohort	Poland	NK^i^	NK^i^		55	49 (89)	0	NR	(2–10)^e^	44 of 55 had spirometry: 35% restrictive pattern, 17% airway obstruction. 13% of cohort were rehospitalised for respiratory illness, 35% had exercise limitation.
Chang et al. [34]^b^	Follow-up of hospitalised cohort	Australia (Aboriginal children)	2000–01	2001–02	Clinical with CXR confirmation (alveolar changes) aged <14 yrs	109	88 (81)	0	NR	1	37.5% with persistent CXR abnormality or respiratory symptoms, 62.5% without these features.

CI, confidence interval; CXR, chest radiograph; FEV_1_, forced expiratory volume in one second; FVC, forced vital capacity; OR, odds ratio; NK, not known; NR, not reported; pred, predicted; UK, United Kingdom

^a^severe lower respiratory tract infection according to maternal report, family physician or study team

^b^Included in review by Thomson et al. [17]

^c^Controls from same gender and class as index child

^d^Mean ± Standard Deviation

^e^Range

^f^Included in review by Edmonds et al. [18]

^g^Only 83 of the 190 cases could be traced

^h^Gender and age matched controls

^i^Unable to obtain article in Polish (data from abstract)

In contrast, two other birth cohorts [26,27] indicated pneumonia in early childhood was associated with an obstructive rather than restrictive lung function deficit (Table 1). The first study was from Derbyshire County, England, where records for babies born between 1917 and 1922 were preserved [26]. These records also included their respiratory health, documented by health visitors in the first 2 years of life. Overall, 618 of the 1,909 (32%) men and women known to have been born in the six chosen districts at that time, now aged in their mid-60s and 70s, completed health questionnaires and underwent spirometry. After adjusting for age, height, smoking, and asthma, the 13 men with pneumonia diagnosed before age 2 years had significantly lower forced expiratory volume in one second (FEV_1_), forced vital capacity (FVC), and FEV_1_/FVC values than the 315 men without a history of pneumonia. The second report was from the Tucson Children’s Respiratory Study, United States of America, (USA) where 1,246 unselected healthy infants were enrolled from 1980 to 1984 and followed into adulthood [27,35]. It reported on those with a history of clinically diagnosed and radiographically confirmed pneumonia during the first 3 years of life [27]. In more than half of the original 66 cases, pneumonia was associated with respiratory viruses (mainly RSV) and in almost two-thirds wheezing was present. None were hospitalised. Forty-four (67%) of these children were followed as adults into their mid-to-late 20s and as a group they had persistently impaired lung function with decreased FEV_1_/FVC ratios and reduced mid-expiratory flow rates indicating small airway obstruction, which was only partially corrected by bronchodilators. Compared to those without pneumonia, these young adults had a two-fold increased risk of active, physician-diagnosed asthma (OR 1.95 [95% CI 1.11–3.44]).

Other birth cohort studies (Table 1) have also reported reduced lung function in adults following childhood lower respiratory tract illnesses. The Newcastle Thousand Families Study, UK, involved 1,142 infants born in 1947 and their lower respiratory illnesses were recorded by health visitors during the first 5 years of life [30]. Of the 252 (22%) who were later recruited from this birth cohort into a nested case-control study in 1961 and underwent spirometry at age 14-years, 122 returned for additional assessments at 49–51 years of age (Table 2). Those with a history of lower respiratory illnesses before age 5-years had significantly lower FEV_1_ recordings than their healthy peers at age 14-years and they also demonstrated a greater decline in FEV_1_ between 14 and 50 years of age. Finally, the Barry Caerphilly Growth Study undertaken in Barry and Caerphilly, Wales, UK, reported the results of spirometry performed on 679 of the original 951 (71%) 25-year-old subjects born in these two towns between 1972 and 1974 and from whom research personnel had gathered information on upper and lower respiratory tract infections during 14 home visits from birth until the subjects reached 5 years of age [28]. The major findings from this study were that lower respiratory tract infections during the first year of life were associated with decreased lung function, involving all lung function parameters except FVC, suggesting these children were at increased risk of airway obstruction. Indeed, a dose-response effect was observed and the authors estimated that a two-fold increase in lower respiratory tract infections during the first year of life was associated with a decrease in FEV_1_ equal to that seen with cigarette smoking for 10–17 pack-years.

### 2.2 Retrospective studies

The review by Edmonds and colleagues [18] identified seven retrospective studies published between 1971 and 2000 that reported outcomes for specific pathogens: adenovirus [36,37], *Mycoplasma pneumoniae* [38–40], *Chlamydia trachomatis* [22], and *Staphylococcus aureus* [41]. However, these data cannot be extrapolated to ‘general pneumonic illnesses’ because of the likely high selection bias inherent in such retrospective studies. Moreover, none of the studies had searched systematically for other co-existing respiratory pathogens that may also have contributed to the child’s clinical presentation and long-term outcomes.

Many other retrospective studies relating childhood respiratory outcomes to poorer adult respiratory health have been published and these include population-based studies, such as that of Barker and Osmond [42], which concluded that there was strong evidence of a direct causal link between acute lower respiratory infection in early childhood and chronic bronchitis in adult life. Furthermore, this study suggested that infection in early childhood had a greater influence than cigarette smoking in determining the geographical distribution of chronic bronchitis with national time trends reflecting the influence of both factors [42]. Most retrospective studies are consistent with the longitudinal studies’ findings that early childhood lung infections are associated with future lung disease or poorer lung function in adults [17,18,26–29].

### 2.3 Studies focused on bronchiectasis only

The rarer complication of bronchiectasis is characterised by chronic wet or productive cough, periodic infectious exacerbations, and irreversible bronchial dilation and wall thickening seen on either plain radiographs or computed tomography of the chest [43]. It normally has multiple aetiologies, including prior severe infections, immune deficiencies, and primary ciliary dyskinesia, and its pathogenesis remains poorly understood. However, bronchiectasis in children from indigenous communities and developing countries is associated with preterm delivery and episodes of early and recurrent pneumonia during infancy [44–47], while 30–60% of adults with recently diagnosed bronchiectasis report repeated lower respiratory tract infections during childhood [48–50]. It is hypothesised that severe pneumonia, including that resulting occasionally from adenovirus outbreaks, can damage ciliated airway epithelium in the growing lung, impairing airway clearance defences and setting up a cycle of repeated or persistent infection and inflammation involving airway infiltration by activated neutrophils and CD4+ T-lymphocytes, followed by degradation of bronchial wall supporting structures, bronchial dilatation, and ultimately bronchiectasis [51]. Nevertheless, studies that identified severe respiratory infections during childhood as risk factors for bronchiectasis were also retrospective and relied upon patient recall or involved highly specific patient groups [48–50]. Readers are referred to the review by Thomson et al [17] that included studies specifically relating to bronchiectasis post-pneumonia.

## 3. Possible explanations for long-term effects

### 3.1 Early lung growth and development

During normal fetal development and the first 3–4 years of rapid post-natal growth, the developing lungs are most vulnerable to injury by infectious and non-infectious insults, including reprogramming by various gene—environment interactions [52,53]. It is during the early post-natal period, when new alveoli are still forming and post-natal lung growth is most rapid, that the developing lungs are most susceptible to the long-term effects of pneumonia. Lung development occurs over five phases, the first four of which are *in utero*, and all five phases can be influenced by maternal, genetic, and environmental factors, including infection in the critical post-natal growth and alveolarisation period [54,55]. Within the developing embryo the lung buds first appear between 4 and 7 weeks, the conducting airways are established by 17 weeks and the respiratory zone, which includes the respiratory bronchioles and alveolar ducts, is fully developed by 27-weeks gestation. Alveolar sacs first emerge, followed by alveoli, after 28 weeks and at full term 100–150 million alveoli have formed. After birth the alveoli continue to multiply reaching the estimated adult number of 300–600 million by at least 3–4 years of age. The alveoli also increase in volume and the airways keep growing, doubling their size by the end of adolescence in females and the mid-twenties for males [55]. During the intrauterine period and the first few years of life various environmental insults to the developing lung may adversely impact upon future respiratory health. For example, preterm delivery can interrupt lung development with subsequent maturation occurring out of phase compared with those born at full term, and the resulting airways are smaller with fewer alveoli and potential negative influences upon lung function [52,56].

Respiratory infections with consequent inflammatory responses may interrupt the critical alveolarisation phase of lung development, restricting alveolar numbers and/or size and leading to often mild, but impaired lung growth. The association between pneumonia and obstructive lung disease is possibly through similar mechanisms to those leading to bronchiectasis, which is an obstructive lung disease. Lung infections at the peak periods of somatic lung growth (a normal newborn has only approximately 5% of a healthy adult’s alveolar surface area [57]) may also alter the programming of lung development at a local or systemic level. The effects of early infection, especially viral lower respiratory tract infections, upon lung growth, programming and future lung function disease types are beyond the scope of this review and readers are referred to the respiratory literature [55,56,58].

### 3.2 Other confounders and effects

In addition to the effects of lower respiratory infections, maternal smoking, and other maternal factors, nutrition, and allergic sensitisation can have potential negative influences upon lung function [52,56,59,60]. Of the above factors, solid experimental evidence on the impact of poor nutrition [61,62] and tobacco smoking in animals [63–65] support the association studies in human literature where infants who are poorly nourished or exposed to tobacco smoke (both *in utero* and in early life) are more likely to have lung infections and are also independently more likely to have adult lung disease and/or poorer lung function [53,55,56]. In animal studies these effects are reversible (at least partially) since improvement in nutrition early in life results in better adult lung development [66]. Pre- and post-natal tobacco exposure have independent long-term pulmonary effects [67,68] and cessation of tobacco exposure improves the accelerated decline in lung function [69]. While it is beyond the scope of this review to elaborate upon these factors (see reviews [60,66,67]), readers should be cognisant of the importance of these issues. The potential reversibility of impaired lung growth and function also highlights the opportunities for future intervenions.

An alternative interpretation is that, irrespective of external factors, lung function from infancy tracks throughout childhood into adult life meaning those predisposed to COPD, bronchiectasis, and other adult lung dysfunction are destined to do so, with pneumonia simply being a biomarker for those with underlying abnormal lung function and growth. Consequently, it follows that no interventions will alter the future development of adult lung disease. This rather nihilistic view is, however, unduly pessimistic. Although randomised controlled trial evidence in humans is not available (and likely never will be), there are many biological reasons why this opinion is incorrect. Examples include marked improvements in survival and lung function seen in patients with cystic fibrosis over the last two decades [70], the reversibility (at least partially) seen in animal studies described above [60,66], and the reduction in mortality from pneumonia through improved case management [7]. Indeed, it is highly likely that the interactions are complex since the host’s respiratory microbiome, immune responses, epigenetic influences, and their physical environment are being recognised increasingly as playing a role in the human phenotypes of health and disease [71]. As shown recently for RSV in unselected term infants [72], both possibilities may not be mutually exclusive. Thus infants with smaller airways and/or reduced numbers of alveoli at birth could have greater numbers of lower respiratory tract infections, including pneumonia, during the critical post-natal period and these infections might also lead to further disruption of alveolar multiplication and/or airway growth and development.

At this point it is also worth noting that recent studies report up to 40% of adults with a chronic productive cough and ‘difficult to control’ asthma may have radiographic evidence of underlying bronchiectasis [73], while 29–58% of those with severe COPD also have bronchiectasis as either a direct complication of COPD or part of an overlap syndrome [74,75]. These reports highlight the complex relationships observed between early childhood respiratory infections and chronic airway disorders in adults. There may be at least two different processes operating in these circumstances producing either functional (COPD) and/or structural (bronchiectasis) defects. A clue to the presence of these mechanisms was provided by a recent prospective study of respiratory exacerbations in infants with cystic fibrosis detected by a newborn screening program [76]. The overall exacerbation rate in the first 2 years of life was negatively associated with FEV_1_ at 5 years of age, suggesting airway remodelling, while only the severe 20% of exacerbations requiring hospitalisation were associated with structural airway injury causing bronchiectasis at age 5-years.

### 3.3 Case ascertainment issues

An important limitation for many studies was the reliance upon parental reporting of symptoms and that some of the cases may have had bronchiolitis, virus-induced wheezing or asthma rather than pneumonia. Even when the diagnosis of pneumonia is supported by radiographic evidence it can still be difficult to differentiate between patchy pulmonary infiltrates and localised atelectasis from mucous plugs in non-hospitalised children with moderate, and often viral, lower respiratory tract illnesses [27]. Radiographic abnormalities (including alveolar consolidation) are often present in infants with moderate-to-severe bronchiolitis [77] and many of the studies relating lower respiratory infections to long-term outcomes do not report on, or do not exclude, presence of wheeze in the study population. The respiratory physiology of wheeze in infants, which is more likely to occur when abnormally small airways and/or atopic sensitisation are present, is probably going to be different to that of pneumonia, which is primarily a parenchymal illness. Consequently, as these diseases have different underlying pathologies and pathogenetic mechanisms, they are also very likely to have different long-term outcomes [59,78,79].

## 4. Studies strengths and weaknesses

The major strengths of the community-based birth cohort studies are the recruitment of unselected subjects and their longitudinal nature spanning several decades, which allowed prospectively collected infant and early childhood exposure data to be analysed with those describing adult respiratory outcomes [23–28,30]. Important limitations though are losses to follow-up, which included those who had died shortly after discharge from hospital [32] and from chronic pulmonary disorders, such as COPD in the older aged cohorts [23,42], as well as those who were too unwell to attend or to perform satisfactory lung function tests. One study assessed only men [23], while those lost to follow-up were usually socioeconomically disadvantaged and may have been at greater risk of respiratory morbidity, even if they had never smoked [80,81]. The early birth cohorts were also conducted before antibiotics were available. Thus the nature or likelihood of injury to the developing lung during alveolarisation may differ whether or not cases of bacterial pneumonia received antibiotics.

Despite the birth cohort studies being prospective, the respiratory illness data were generally collected by visiting healthcare workers, usually some weeks or months later, and in one study up to 7 years after the event [25]. Consequently, the diagnosis of pneumonia often relied upon parental recall and in only one of these community-based studies was the diagnosis of pneumonia made by a paediatrician and confirmed radiographically [27]. Moreover, most studies provided little information on the number of pneumonia episodes experienced at an individual level, limiting our capacity to determine if a single episode in an infant or young child is associated with impaired lung function in later adult life or if instead pneumonia needs to be severe or recurrent to have such effects. In addition, the subjects in some studies were asked to self-report some potentially important confounding variables, such as gestation at delivery, maternal smoking, and occupational exposures to environmental toxicants. As discussed above, an unknown proportion of these episodes may have been from bronchiolitis, virus-induced wheeze, or asthma rather than pneumonia. Thus, these studies suffered from varying degrees of potential selection, gender and recall bias, as well as diagnostic misclassification. Also, as with any observational studies, the possibility of residual unmeasured confounding remains an issue.

The hospital-based studies reported in the systematic review [18] involved small numbers of subjects (20–190), of whom 19–59% were followed for a mean of 7.8 (range 1.5–17) years. All those reporting upon a single respiratory pathogen (*S. aureus, M. pneumoniae* and *C. trachomatis*) were retrospective. Each study was limited by incomplete data collection, failure to use sensitive diagnostic techniques to exclude co-infection by other respiratory pathogens, likely referral bias, and differential patient follow-up where the potential existed for children with symptoms to be more likely to be brought back for review. Finally, other major limitations were the lack of baseline clinical and lung function data prior to the episode of pneumonia in all but one study [27] and the lack of data on potential confounders and the effect of interventions provided.

## 5. Conclusions and future directions

It is widely recognised that pneumonia in young children causes a considerable worldwide burden of mortality and short-term morbidity in survivors. What is less well known is that infectious insults to the rapidly growing and still developing lungs in the first 1–3 years of life are independently associated with an increased risk of impaired lung function in adulthood. The risks appear greatest for those whose illness is of sufficient severity to warrant treatment in hospital. The long-term effects associated with early childhood pneumonia include restrictive or obstructive lung function deficits and an increased risk of adult asthma, non-smoking related COPD, and bronchiectasis. The studies underpinning these observations do however have important limitations. They are a mixture of prospective and retrospective studies, involving both community- and hospital-based populations experiencing illness of varying severity, with incomplete follow-up and opportunities for sampling and recall bias, and diagnostic misclassification of bronchitis, bronchiolitis, viral-induced wheezing and asthma as pneumonia. Most important of all is that most studies do not have prior lung function data for their pneumonia cases and subsequent impairments in lung function might simply reflect pre-existing abnormalities in already susceptible infants and young children.

More high-quality, long-term studies are needed, ideally involving birth cohorts from both developing and developed countries with antenatal recruitment and, whenever feasible, measurements of lung function should be conducted shortly after birth. To minimise the risk of misclassification, standardised diagnostic criteria for pneumonia should also be used. Such studies are challenging and expensive to perform, but can help focus future research, while helping to guide clinical practice and child public health policies. A promising start has been made in South Africa with the Drakenstein Child Health Study [82]. Although, by relying solely upon the World Health Organization clinical definitions of pneumonia many of the cases will have lower airway, rather than alveolar involvement, and thus several of the questions raised in this review will not be addressed by this birth cohort study. The underlying airway cellular, immunological, and microbiological mechanisms associated with chronic pulmonary disorders originating in the antenatal and early childhood periods are also important, but beyond the scope of this review and are discussed elsewhere [54–59,83]. However, gaining an understanding of these mechanisms is vital for developing effective therapeutic interventions to either prevent or reverse lifelong injury to the developing lungs. Meanwhile, for clinicians it is important to recognise that young children with pneumonia are at risk of chronic pulmonary disease as adults, irrespective whether this is a direct result of their infection or that susceptibility to pneumonia itself is a marker for potential underlying deficits in lung function. Either way, a careful history should be taken for each patient, recording details of the pregnancy, nature and timing of the birth, maternal and household tobacco smoking history, exposure to indoor (e.g. biomass fuels) and outdoor air pollution, prior history of respiratory symptoms, especially chronic cough, wheezing or breathing difficulties, previous respiratory illness episodes, their nutritional state, immunisation record and personal and family histories of atopy, asthma or other chronic lung diseases. The parents and caregivers should be counselled about the possibility of adult-onset lung disease in the child and advised accordingly of the importance of avoiding active and passive smoking, indoor and outdoor air pollution, future occupational inhalant exposures, and ensuring that all recommended vaccines are received in a timely manner. Public health interventions, such as appropriate housing, household heating and hygiene, reducing barriers to accessing healthcare and immunisation services, promoting education, and improving socio-economic status are also important [29,84], especially in developing countries and the economically disadvantaged in developed countries (e.g. indigenous populations in Australia, New Zealand, Canada, and the USA with high burdens of lung disease [85]).

Finally, it is essential that the complex relationships between pre-existing lung impairment, early childhood pneumonia, and chronic pulmonary disorders in adults are disentangled and better understood so that policy makers can make informed decisions about public health interventions to help stem the tide of chronic lung disease in adults. As an example, it will be necessary to take into account that vaccines against common respiratory pathogens may have longer-term benefits than simply protecting against episodes of acute pneumonia. By 2030, COPD is predicted to become the third leading cause of deaths globally [86], and with almost half of the cases now occurring in adults who have never smoked, the roles played by exposure to other inhaled environmental toxicants and lower respiratory tract infections during early childhood need to be highlighted, investigated, and managed at both an individual and population level [87].
